# Supplementary material for: Development of Antibodies against HPV-6 and HPV-11 for the Study of Laryngeal Papilloma
Source: Viruses. 2021 Oct 7;13(10):2024. doi: 10.3390/v13102024 (PMC8539961; doi:10.3390/v13102024)
Supplement: Supplementary file 1 [file viruses-13-02024-s001.zip › viruses-1342356-supplementary.pdf]

Supplementary Materials for

Development of Antibodies against HPV-6 and HPV-11 for the Study of Laryngeal Papilloma

Contents

Table S1. Primers used in the present study

Table S2. Target gene, method, standard DNA, detection range, and amplification efficiency in real-time PCR

Supplementary Methods

Table S1. Primers used in the present study

**Cloning primers used for standard HPV-11 DNA**	**Sequence (5′–3′)**
HPV-11 F1	AGCAGACGAGGCATTATGGA
HPV-11 R1	AGAAACTGCACAATTGGCTTCC
HPV-11 F2	ACTGGAAGCCAATTGTGCAGT
HPV-11 R2	CACCATTATTGTTGCGTTTGCAC
HPV-11 F3	TCTGTGCTGGTACTAACACTTCT
HPV-11 R3	CCTGTGCACGCCCATACTAA
**Real-time PCR primers and TaqMan probes**	**Sequence (5′–3′)**
HPV-11 E6-F	TAAAGATGCCTCCACGTCTGC
HPV-11 E6-R	CGGTGGTCAGTGCATTCCT
HPV-11 E7-F	AGCTCAGAAGATGAGGTGGAC
HPV-11 E7-R	TCACATCCACAGCAACAGGTC
HPV-11 E1-F	GCGGATGCTCATTATGCGAC
HPV-11 E1-R	AATGGCGTCTAACCGTGGAC
HPV-11 E2-F	GAGCCACATTGGGCTACAAG
HPV-11 E2-R	ATGTCCAAGGTTCCACACCAT
HPV-11 E4-F	CTACTACATACACCCCCGCAC
HPV-11 E4-R	ACAGGGTGTTGTTAGTGGACG
HPV-11 E5a-F	AGTGCCTGTACAAATTGCTGC
HPV-11 E5a-R	AGTGTTAGTACCAGCACAGATG
HPV-11 E5b-F	ACATGGTTGTTTCTGTGGTTGT
HPV-11 E5b-R	TTCAGTACCATGTACAGCCCT
HPV-11 L2-F	GGTCCATGTACACACGCAGT
HPV-11 L2-R	AGCCACTAGAGGGTGCAGTT
HPV-11 L1-F	GCCTAGCGACAGCACAGTAT
HPV-11 L1-R	ATGTCCCACAGCAAGGAGTC
β-Globin-F	TGGGTTTCTGATAGGCACTGACT
β-Globin-R	AACAGCATCAGGAGTGGACAGAT
β-Globin-probe	FAM-TCTACCCTTGGACCCAGAGGTTCTTTGAGT-TAMRA
β-Actin-F	GCGAGAAGATGACCCAGATC

β-Actin-R	CCAGTGGTACGGCCAGAGG
β-Actin-probe	FAM-CCAGCCATGTACGTTGCTATCCAGGC-TAMRA
**Primers for ISH probes**	**Sequence (5′–3′)**
HPV-11 E6-ISH-F	ATGGAAAGTAAAGATGCCTCCAC
HPV-11 E6-ISH-R	TCCAGCAGTGTAAGCAACGAC
HPV-11 E2-ISH-F	ATGGAAGCAATAGCCAAGC
HPV-11 E2-ISH-R	CATACCACATACTCCATTACATTGTC
HPV-11 E4-ISH-F	ATCATTGGGAAGTATGTTATGGC
HPV-11 E4-ISH-R	TATAGGCGTAGCTGCACTGTGAC
HPV-11 E5b-ISH-F	GATAATGGAGATTATGTGTACATGAACTAA
HPV-11 E5b-ISH-R	ATGGTGATGTTAACCTGTCACTTAAAT
**Primers for pET22b (+) E1^E4 expression vector**	**Sequence (5′–3′)**
HPV-11 E1^E4-*Nde*I-F	AAAACATATGGCGGACGATTCAGC
HPV-11 E1^E4-*Bgl*II-F	AAAAAAGATCTGCGGACGATTCAGCACTGT
HPV-11 E1^E4-*Bam*HI-*Xho*I-R	TTTTTCTCGAGGCCACCGGATCCTAGGCGTAGCTGCACTGTGA

Table S2. Target gene, method, standard DNA, detection range, and amplification efficiency in real-time PCR

**Target gene**	**Method**	**Standard DNA**	**Detection range**	**Amplification efficiency (%)**
HPV-11 E6	SYBR Green	Clone A	$2.0 \times 10^1 – 2.0 \times 10^7$ copies	102.2
HPV-11 E7	SYBR Green	Clone A	$2.0 \times 10^3 – 2.0 \times 10^7$ copies	107.6
HPV-11 E1	SYBR Green	Clone A	$2.0 \times 10^1 – 2.0 \times 10^7$ copies	115.3
HPV-11 E2	SYBR Green	Clone B	$2.0 \times 10^1 – 2.0 \times 10^7$ copies	101.8
HPV-11 E4	SYBR Green	Clone B	$2.0 \times 10^1 – 2.0 \times 10^7$ copies	103.5
HPV-11 E5a	SYBR Green	pBR322.HPV11 (ATCC no. 45151)	$1.0 \times 10^2 – 1.0 \times 10^7$ copies	102.0
HPV-11 E5b	SYBR Green	Clone C	$2.0 \times 10^1 – 2.0 \times 10^7$ copies	109.2
HPV-11 L2	SYBR Green	Clone C	$2.0 \times 10^1 – 2.0 \times 10^7$ copies	109.5
HPV-11 L1	SYBR Green	Clone C	$2.0 \times 10^1 – 2.0 \times 10^7$ copies	104.2
β-Actin	TaqMan probe	pCAG-mGFP-actin	$10^1–10^7$ copies	108.2
β-Globin	TaqMan probe	Human placental DNA	0.3–300 ng	90.1

Supplementary Methods

Detection of HPV DNA

DNA was extracted from fresh-frozen samples using a Gentra Puregene Tissue Kit (Qiagen, Gaithersburg, MD) [11,15]. The primers PC04 and GH20 were used to determine the presence and integrity of DNA in all samples by PCR amplification of the β-globin gene.

A negative control (water) and a positive control (DNA from an HPV-16-positive CaSki cell line) were included in each amplification series. We used the degenerate consensus primer sets GP5+/GP6+ and MY09/MY11 [11], which were designed to amplify the *L1* region, to determine the HPV subtypes in the samples. When no PCR amplification occurred with the GP5+/GP6+ or MY09/MY11 primers, 10-fold diluted first-round PCR products were used as template DNA for nested PCR using the GP5+/GP6+ primer pair. PCR products of the expected size (GP5+/GP6+, 150 bp; MY09/MY11, 450 bp) were purified and directly sequenced with an ABI PRISM 3130xl Genetic Analyzer (Applied Biosystems, Foster City, CA). The sequences were aligned and compared with the *L1* gene of known HPV subtypes in GenBank using BLAST (https://blast.ncbi.nlm.nih.gov/Blast.cgi).

Measurement of Viral DNA Load and mRNA Expression in HPV-11-infected Papilloma by Quantitative Real-time PCR

Total RNA was extracted from fresh-frozen papilloma samples with RNAiso Plus (Takara Bio Inc., Otsu, Japan) according to the manufacturer's instruction. Total RNA (500 ng) from each sample was reverse-transcribed using the PrimeScript® RT Reagent with gDNA Eraser (Takara). To establish real-time PCR assays to measure the absolute levels of *E6*, *E7*, *E1*, *E2*, *E4*, *E5a*, *E5b*, *L2,* and *L1* mRNAs, three regions of HPV-11 were cloned using genomic DNA from Patient 1 (clones A, B, and C in Figure 1). Clone A was amplified from the anterior region of the *E6* gene to the posterior one-third of the *E1* gene (nt87–nt2167, 2,005 bp) using the F1 and R1 primers (Table S1 in the Supplementary Materials). Clone B was amplified from the posterior one-third of the *E1* gene to the anterior one-third of the *E5b* gene (nt2147–nt4151, 2,005 bp) using the F2 and R2 primers (Table S1 in the Supplementary Materials). Clone C was amplified from the anterior half of the *E5a* gene to the anterior one-third of the *L1* gene (nt4003–nt6072, 2,070 bp) using the primers F3 and R3 (Table S1 in the Supplementary Materials). The PCR reaction mixture (12.5 μL) contained 1 μL genomic DNA from Patient 1 (30 ng/μL), 0.24 μM forward and reverse primers, and 6.3 μL GoTaq® Green Master Mix (Promega, Madison, WI). PCR was performed under the following conditions: 95°C for 15 min, followed by 40 cycles at 95°C for 30 s, 55°C for 30 s, and 72°C for 3 min, and finally 72°C for 7 min. The PCR fragments of the expected size were purified using the Wizard SV Gel and PCR Clean-Up System (Promega). The purified PCR products were cloned into the pGEM-T Easy vector (Promega) and sequenced using an ABI PRISM 3130xl Genetic Analyzer. The sequences obtained were analyzed as aforementioned. The plasmid of clone B in the present study contained a 241-bp deletion of the *E5a* gene (Figure 1). Thus, the pBR322.HPV11 plasmid, deposited as ATCC no. 45151, which encodes the *E5a* gene [29], was serially diluted from 1.0×10^2 to 1.0×10^7 copies/2 μL to establish a standard curve for the *E5a* gene in real-time PCR. Similarly, the plasmids (clones A, B, and C) were serially diluted from 2.0×10^1 to 2.0×10^7 copies/2 μL to generate standard curves for the *E7*, *E1*, *E2*, *E4*, *E5b*, *L2,* and *L1* genes, as described above. Real-time PCR was performed with the ABI Prism 7300 Sequence Detection System (Applied Biosystems) or CFX96 Touch™ Real-Time PCR Detection System (Bio-Rad, Hercules, CA). The PCR reaction mixture (10 μL) contained 0.2 μM primers, 5 μL SYBR Premix Ex Taq™ II (Tli RNaseH plus; Takara), and 2 μL standard plasmid DNA (from 2.0×10^1 to 2.0×10^7 copies or from 1.0×10^1 to 1.0×10^7 copies), genomic DNA (30 ng/2 μL), or cDNA (20 ng/2 μL). PCR was performed under the following conditions: 95°C for 30 s

followed by 40 cycles at 95°C for 5 s and 60°C for 30 s. Specific amplification of each cDNA was verified by melting curve analysis and gel electrophoresis of the PCR products. To quantify sample DNA amounts, an external standard curve was created using known serial dilutions (0.3, 3, 30, and 300 ng) of human placental genomic DNA (Sigma-Aldrich, St. Louis, MO), and β-globin was amplified as described previously [12]. The amplification efficiency of target genes is shown in Table S2 in the Supplementary Materials. Viral load was defined by *E6* copy number/ng cellular DNA. β-Actin mRNA was measured as an internal control for sample cDNA by a standard curve (from 1.0×10^1 to 1.0×10^7 copies) as described previously [11]. β-Actin mRNA levels were used to normalize all viral mRNA levels.

ISH with HPV DNA Probes

Biotinyl tyramide-based ISH was performed using the HPV types 6/11 biotinylated DNA probe (Y1411; Agilent Technologies, Inc., Santa Clara, CA) and GenPoint tyramide signal amplification system for biotinylated probes as described previously [11]. Briefly, serial 4-μm-thick sections of formalin-fixed paraffin-embedded (FFPE) samples were deparaffinized in xylene and rehydrated using a graded alcohol series. Target HPV DNA retrieval was performed in 10 mM sodium citrate (pH 6.0) at 95°C. The slides were digested with proteinase K, and endogenous peroxidase activity was blocked with 0.3% H_2O_2 in methanol. A drop of the HPV probe was added to the section, and a coverslip was applied. The probe and target DNA were denatured by incubating the slides at 92°C. After denaturation, the slides were transferred to a humidified chamber for hybridization at 37°C for 16 h. The slides were washed using GenPoint Detection system stringent wash solution (Agilent Technologies) at 48°C for 30 min, followed by rinsing in Tris-buffered saline containing 0.05% Tween 20. Detection of the hybridized probe was performed using the GenPoint Detection system with primary streptavidin-horseradish peroxidase (HRP), biotinyl tyramide, secondary streptavidin-HRP, and 3-3′-diaminobenzidine (Agilent Technologies). The slides were counterstained with hematoxylin.

RNA-ISH with HPV-11 E6, E2, E4, and E5b Digoxigenin RNA Probes

For RNA-ISH, the *E6*, *E2*, *E4*, and *E5b* genes of HPV-11 were amplified by the following PCR method (Figure 1) using the primer sets shown in Table S1 in the Supplementary Materials. The PCR reaction mixture (12.5 μL) contained 1 μL template plasmid (clone A plasmid for *E6*; clone B plasmid for *E2* and *E4*; clone C plasmid for *E5b*), 0.24 μM forward and reverse primers, and 6.3 μL GoTaq® Green Master Mix (Promega). PCR was performed under the following conditions: 95°C for 5 min, followed by 35 cycles at 95°C for 30 s, 60°C for 30 s, and 72°C for 1 min, and finally 72°C for 5 min. The PCR products were subcloned into the pGEM-T Easy vector (Promega) as described above. Anti-sense RNA probes were transcribed from linearized plasmids using a digoxigenin (DIG)-labeling mix (Roche Diagnostics, Mannheim, Germany) and SP6 or T7 RNA polymerase (Takara) and then digested with DNase I (Takara). A total of 1 μg linearized plasmid DNA was used in a 20-μL reaction for DIG-RNA labeling. The probes were stored at −80°C until use.

HPV-11-infected LP FFPE samples were sectioned at 4 μm and mounted on adhesive glass slides (Platinum Pro®; Matsunami Glass, Osaka, Japan). RNA-ISH was conducted as described previously [11]. Briefly, the sections were deparaffinized in xylene and rehydrated in a graded alcohol series, followed by incubation in ultra-pure water. After washing with phosphate-buffered saline (PBS) with 0.1% Tween 20 (PBST), the sections were incubated in PBST including proteinase K (final concentration 0.01 mg/mL; Agilent Technologies) at 37°C and fixed in 4% paraformaldehyde (Nacalai Tesque, Kyoto, Japan) at room temperature. After washing with PBST, pre-hybridization was performed in a

hybridization buffer containing 50% deionized formamide, 5× saline-sodium citrate (SSC), 50 μg/mL heparin (Nacalai Tesque), 100 μg/mL *Escherichia coli* tRNA (Roche Diagnostics), 1% sodium dodecyl sulfate (SDS), and 0.1% Tween 20 at 60°C for 1 h. Hybridization was performed with DIG-labeled RNA probes (1 μg/mL in hybridization solution) at 60°C overnight in a humidified chamber. After hybridization, the sections were washed as follows: for 5 min in 50% formamide in 2× SSC with 0.1% Tween 20 at 60°C; 5 min in 2× SSC at 60°C; and 5 min in 0.2× SSC at room temperature. The sections were washed three times with PBST at room temperature, incubated with blocking buffer (0.5% blocking reagent in PBST; Roche Diagnostics), and incubated with 1:3,000 anti-DIG-alkaline phosphatase (Roche Diagnostics) in the blocking buffer at 4°C overnight. The sections were rinsed three times with PBST and washed with reaction buffer (100 mM Tris-HCl, 100 mM NaCl, 50 mM $MgCl_2$, pH 8.0). Section detection was carried out using a nitroblue tetrazolium/5-bromo-4-chloro-3-indolyl phosphate solution (Roche Diagnostics) in the reaction buffer. The sections were washed three times with PBST and counterstained with Vector® Nuclear Fast Red (Vector Laboratories, Burlingame, CA).

Western Blotting

HEK293T cells were transfected with HPV-6 E1^E4, HPV-11 E1^E4, or human CMTM7 genes (pcDNA3.1+ HPV-6 E1^E4-3× FLAG, pcDNA3.1+ HPV-11 E1^E4-3× FLAG, or pcDNA3.1+ CMTM7-3× FLAG) as described previously [11] to evaluate the antibodies against HPV-11 E1^E4 and HPV-6 E1^E4. The cells were cultured for 48 h after transfection. Mock-transfected cells (empty pCDNA3.1+) were also included in the transfection experiment. HEK293T cells expressing HPV-6 *E1^E4*, HPV-11 *E1^E4*, or human *CMTM7* and mock-transfected HEK293T cells were lysed in a buffer containing 62.5 mM Tris-HCl (pH 6.8), 2% SDS, and 10% glycerol. Lysate protein concentration was measured using a DC Protein Assay Kit (Bio-Rad). Each protein sample was prepared at 50 and 10 μg/10 μL in 62.5 mM Tris-HCl (pH 6.8), 2% SDS, 10% glycerol, 6% mercaptoethanol, and 0.01% bromophenol blue and boiled for 5 min. The samples were separated by 12.5% SDS-polyacrylamide gel electrophoresis (PAGE) and transferred to polyvinylidene difluoride membranes.

FLAG protein was detected with an HRP-conjugated monoclonal anti-FLAG M2 antibody (Sigma-Aldrich) diluted at 1:1,000 with Can Get Signal Solution 2 (Toyobo, Osaka, Japan) to confirm the expression of HPV-6 E1^E4, HPV-11 E1^E4, and human CMTM7. The antiserum against HPV-11 E1^E4 was diluted at 1:5,000 with Can Get Signal Solution 1 (Toyobo) to evaluate the specificity of the rabbit anti-HPV-11 E1^E4 polyclonal antibody. A secondary antibody, goat IgG-conjugated with HRP against rabbit IgG1 (Cell Signaling Technology, Danvers, MA), was used at a dilution of 1:1,000 with Can Get Signal Solution 2 (Toyobo). The bands were visualized using Clarity Western ECL Substrate (Bio-Rad) and the ChemiDoc XRS+ System (Bio-Rad).

Immunohistochemistry Using the Newly Developed Anti-HPV-11 E1^E4 Antibody and Anti-HPV-6 E1^E4 Antibody

The sensitivity and specificity of the newly developed anti-HPV-11 E1^E4 antibody and anti-HPV-6 E1^E4 antibody were in immunohistochemistry.

Four-μm-thick sections from FFPE samples obtained from 66 patients were deparaffinized in xylene and hydrated in a graded alcohol series. Epitope retrieval was achieved by heating at 100°C for 10 min in 1 mM EDTA buffer (pH 8.0). Endogenous peroxidase activity was quenched by incubating the sections in 0.3% H_2O_2 in methanol for 20 min at room temperature. A SAB-PO Kit (Nichirei Bioscience, Tokyo, Japan) was used as described previously [11] to detect immunoreactivity to HPV-11 E1^E4. After blocking non-specific reactions by incubation in 10% goat serum, the slides were incubated

with the self-made antibody, rabbit polyclonal anti-HPV-11 E1^E4 antibody (antiserum against HPV-11 E1^E4), or alpaca-mouse monoclonal, the chimeric antibody against HPV-6 E1^E4 [11]. The anti-HPV-11 E1^E4 antibody was diluted at 1:1,000 with Protein Block Serum-Free blocking solution (Agilent Technologies, Inc.), whereas the anti-HPV-6 E1^E4 antibody was used without dilution. The tissue slides were incubated with the primary antibodies overnight at 4°C. Subsequently, a biotin-labeled secondary antibody and peroxidase-labeled streptavidin were applied. Immunolabeling was visualized by incubation in 3-3′-diaminobenzidine, and stained slides were counterstained with hematoxylin.
